# Development of an HPV Genotype Detection Platform Based on Aggregation-Induced Emission (AIE) and Flow-Through Hybridization Technologies

**DOI:** 10.3390/molecules27207036

**Published:** 2022-10-18

**Authors:** Chun-Ho (Charlie) Ma, Liejun Li, Shuheng Cai, Pei Lin, Wing-Ki (Kristy) Lam, Tsz-Him (Ronald) Lee, Tsz-Kin (Ryan) Kwok, Longxu Xie, Tit-Sang (Tom) Kun, Ben-Zhong Tang

**Affiliations:** 1 Department of Chemical and Biological Engineering, Department of Chemistry, Hong Kong Branch of Chinese National Engineering Research Center for Tissue Restoration and Reconstruction, The Hong Kong University of Science and Technology, Clear Water Bay, Kowloon, Hong Kong, China; 2 Guangzhou Hybribio Biotech Limited, No 71, Fenghuang 3rd Road, Sino-Singapore Guangzhou Knowledge City, Guangzhou 510000, China; 3 Hybribio Limited, Strand 50, Bonham Strand, Sheung Wan, Hong Kong, China; 4 School of Science and Engineering, Shenzhen Institute of Aggregate Science and Technology, The Chinese University of Hong Kong, Shenzhen 518172, China

**Keywords:** aggregation-induced emission, flow-through hybridization, genetic diseases, alkaline phosphatase, human immunodeficiency virus, membrane reader

## Abstract

Genetic mutations can cause life-threatening diseases such as cancers and sickle cell anemia. Gene detection is thus of importance for disease-risk prediction or early diagnosis and treatment. Apart from genetic defects, gene detection techniques can also be applied to gene-related diseases with high risk to human health such as human papillomavirus (HPV) infection. HPV infection has been strongly linked to cervical cancer. To achieve a high-throughput HPV gene detection platform, the flow-through hybridization system appears to be one of the commercialized diagnostic techniques for this purpose. The flow-through hybridization technique is based on a vacuum-guided flow of DNA fragments which is continuously directed toward the oligoprobes that are immobilized on the testing membrane. However, the conventional colorimetric method and signal read-out approach suffers a problem of low sensitivity. On the contrary, fluorescence approaches allow more sensitive detection and broad sensing ranges. In this work, a fluorescent dye HCAP, which possesses aggregation-induced emission (AIE) properties and is responsive to alkaline phosphatase, was developed and applied to the flow-through hybridization platform to achieve HPV genome diagnosis of clinical samples. Also, an automatic membrane reader was constructed based on the AIE-based diagnosis platform which can identify the diagnostic result of patient DNA with a total concordance rate of 100% in the clinical trial.

## 1. Introduction

Point mutations in specific genes cause life-threatening diseases including cancers and sickle cell anemia, which are among the leading causes of death globally [1]. Gene detection is thus of importance for disease-risk prediction or early diagnosis and treatment. Apart from genetic defects, detection techniques can also be applied to gene-related diseases with high risk to human health such as human papillomavirus (HPV) infection [2]. HPV infection can increase the risk of cancer at the site affected and has been proven to cause cervical cancer [3]. Among the HPV family, HPV16 and HPV18 are the two high-risk (HR-HPV) strains that are detected in most cervical cancer patients [4,5]. Additionally, HPV viral DNA is able to maintain a trace number of viral plasmids in dividing basal cells, which could persistently infect the host with no symptoms and transmits to other hosts through social behaviors [6,7]. Therefore, in many countries, gene detection has been widely applied in the screening of cervical cancer and for preventing the spread of HPV [8].

Undoubtedly, advanced gene detection platforms such as microchip technology have revolutionized genomic and proteomic research, due to their abilities to detect specific gene sequences, proteins, and other analytes [9]. However, the microchips used in detection usually consist of thousands of unique DNA sample spots immobilized on a nickel-coated glass microscope slide with a length of several millimeters [10,11]. In addition, microarray analysis also requires a series of steps including the separation of messenger RNA (mRNA) from patient samples, conversion of mRNA to its complementary DNA (cDNA), and fluorescent labeling of sample cDNA strands [12]. The complicated nature of array technology makes them difficult to apply widely.

Practically, definitive gene diagnosis is usually performed using hundreds or even fewer sequences or analytes collected from available research data. Therefore, a more specific technique with simpler construction is required to achieve a more affordable, efficient, and effective diagnosis for worldwide development. The concept of membrane hybridization was firstly reported by Southern and subsequently modified by Maggio [13,14]. The technique allows rapid binding between polymerase chain reaction (PCR)-amplified DNA samples and oligonucleotide probes coated on the nylon membrane [15]. Taking the advantage of PCR, each single DNA strand can be amplified into millions of copies in a short time, which significantly enhances the sensitivity and specificity of diagnosis for trace DNA content or a very diluted sample [16,17]. Under the optimum clinical conditions, PCR can determine multiple diseases from a single patient sample, and the analysis result of the nucleic acid will generally be available at three levels of resolution. The sequence of genes can precisely identify the genome with the highest resolution, which generates abundant data and is thus preferred by researchers [2,18]. However, the approach is not generally used due to its high cost, time-consuming nature, and requirement for analytical experience. Multiplex analysis is a medium-resolution approach that identifies a specific type of DNA or RNA molecule and its variants with high accuracy [19]. Real-time PCR testing employs radioactive isotopes and isotopic-labeling fluorophores to detect specific genes, which provides immediate test results with the lowest resolution [20]. This approach is widely used in commercial laboratories for disease screening, food content testing, and other commercial applications [21,22].

To develop a commercialized HPV detection kit with high-throughput and improved sensitivity, the flow-through hybridization platform appears to be one of the more effective diagnostic techniques for simplified disease detection [23,24]. The employment of membrane hybridization techniques provides sufficient resolution and accuracy for performing HPV diagnosis with simpler equipment and lowered operation cost. Flow-through hybridization is based on a vacuum-guided flow of DNA fragments which are continuously directed toward the oligoprobes that are immobilized on the membrane. Compared to the conventional membrane hybridization approach, flow-through hybridization improves intermolecular interactions occurring in three-dimensional volumes rather than on the two-dimensional surface, which facilitates the binding interaction and allows more efficient hybridization between the target gene and capture probes. Hybribio Ltd. developed a set of detection kits from sample DNA amplification to flow-through hybridization, to identify gene-related diseases such as HPV, STDs, and thalassemia by using specific capture probes with gene-chip technology. The commercialized platform is a kind of cost-effective and low-density macro-array, which could be a potential competitor to the existing DNA micro-array technology with higher cost and more complicated construction. The flow-through hybridization technology shows superior advantages, including reduced hybridization duration from hours down to minutes, smaller sample and reagent volumes, lower cost, and a simple signal-detection methodology [25].

The colorimetric method is employed currently in the signal read-out approach of Hybribio’s platform. Enzyme-sensitive chromogenic substrates, such as the mixture of 5-bromo-4-chloro-3′-indolyphosphate p-toluidine salt (BCIP) and nitro-blue tetrazolium chloride (NBT) could form intense, insoluble black-purple precipitates after reacting with alkaline phosphatase (ALP) [26]. In the presence of ALP labels, the dye will form colorimetric signals on the boxes to detect disease-related genes in the patient sample. However, colorimetric detection is considered a medium-sensitivity method. To develop a more sensitive detection platform, fluorometric approaches are preferable [27].

Despite the superiority in signal sensitivity, fluorescent dyes usually suffer from strong background signals and weakened emission upon labeling which results in a low signal-to-noise (S/N) ratio [28]. Fluorescein-5-isothiocyanate (FITC) has been widely applied in biological research as a fluorescent reagent due to its strong emission and excellent water solubility [29]. FITC can bind with biomarkers to achieve labeling for different detection purposes [30,31]. After the straining process, the aqueous solution of FITC must be removed by washing due to its strong background. However, the excessive washing process could damage the fluorescent labels which could affect the accuracy and integrity of detection results.

**Figure 1:**
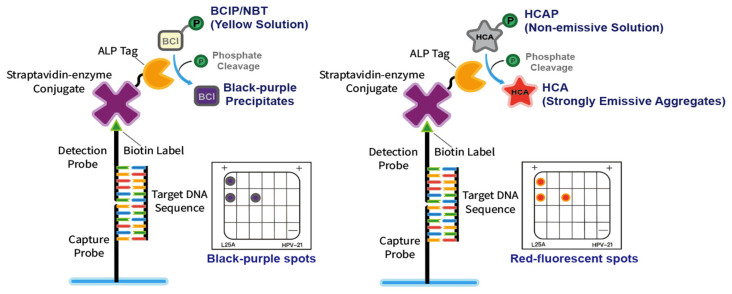
Schematic diagrams of the colorimetric (BCIP/NBT) and the fluorometric (HCAP) approaches for the HPV genotype detection platform.

Luminogens with aggregation-induced emission characteristics (AIEgens) exhibit superiority in signal output and reading [32]. At low concentrations, the propeller-shaped structure of AIEgens undergoes low-frequency motions and dissipates the excitation energy, which leads to fast, non-radiative decay of excited states and weak emission [33]. In the aggregation state, the restriction of intramolecular motion will inhibit non-radiative decay pathways and result in strong fluorescent signals [34]. Through deliciated molecular design, AIE fluorescent probes can be readily functionalized for a variety of biological applications, such as monitoring enzyme activities [35]. HCAP (2-(3-(4-(dimethylamino)phenyl)acryloyl)phenyl phosphate) was reported as an alkaline phosphatase (ALP)- responsive AIEgen [36]. The non-emissive aqueous solution of HCAP readily forms strongly emissive red aggregates in the presence of ALP. The ‘turn-on’ property can reduce background signal and prevent excessive washing steps for conventional fluorescent labels. [36,37] Thus, this kind of ALP-responsive AIE probe could be an ideal substitute for the conventional BCIP/NBT colorimetric system due to its simpler molecular structure, straightforward one-step ‘turn-on’ mechanism, and strong fluorescent emission (Figure 1).

**Figure 2:**
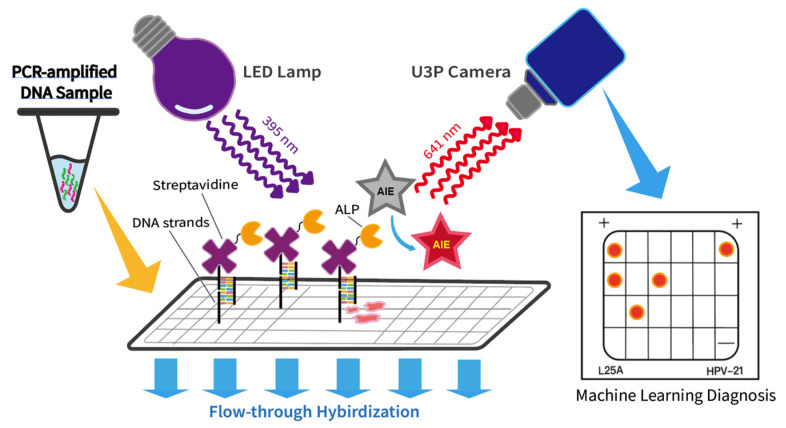
Schematic diagram of the HPV genotype detection platform based on aggregation-induced emission (AIE) and flow-through hybridization technologies.

In this work, we developed an HPV genotype detection platform based on AIE luminogen HCAP and flow-through hybridization technologies (Figure 2 and Figure S1 from Supplementary Materials). The platform can be operated with ease and detect multiple targets in a single run. By optimizing factors such as dye concentration, incubation time, and PCR cycles, the AIE-based detection platform can detect clinical samples with a variant of HPV genotype within a reduced testing time, high accuracy, and high-contrast fluorescent signals. Additionally, an automatic membrane reader was constructed to identify the fluorescent spot data on the testing result and provide an accurate machine-learning diagnosis.

## 2. Results and Discussion

### 2.1. Photophysical Properties of HCA and HCAP

**Figure 3:**
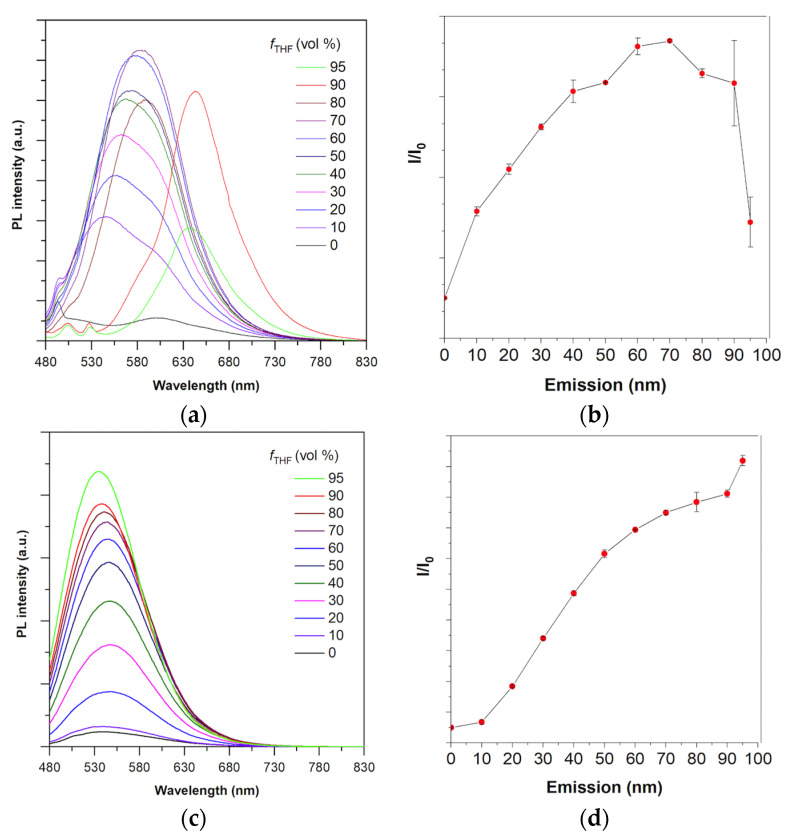
Photophysical properties of HCA and HCAP. (**a**) PL spectra of HCA (10 μM) in THF/water mixtures with different water fractions. (**b**) Plot of relative PL intensity (I/I_0_) versus the composition of the aqueous mixture of HCA. I_0_ = PL intensity in pure THF. (**c**) PL spectra of HCAP (10 μM) in water/THF mixtures with different THF fractions. (**d**) Plot of relative PL intensity (I/I_0_) versus the composition of the aqueous mixture of HCAP. I_0_ = PL intensity in pure water.

The synthetic route to HCAP is depicted in Figure S2 and the detailed synthetic procedure is described in the experimental Session. The absorption maxima of HCA in THF and HCAP in water appeared at 430 nm and 416 nm, respectively. HCA is a hydrophobic fluorescent compound that dissolves well in THF but dissolves poorly in water. Therefore, its AIE property was evaluated by the addition of different fractions of water in its THF solution. As shown in Figure 3a,b, as the water fraction increased from 0% to 70%, the fluorescence intensity of HCA was gradually enhanced. At 90% water fraction, the emission spectrum was red-shifted and the error value of PL intensity increased significantly. The phenomenon could be explained by the random formation of tiny crystals induced by the poor water solubility of HCA. The red-shifted emission of HCA at high water content should be attributed to the reformation of intramolecular hydrogen bonding in HCA, and the ESIPT process leading the keto form of HCA to emit red fluorescence. Similar results were observed and explained in the previous report [36]. When more water was added, the emission was slightly decreased due to the formation and precipitation of amorphous aggregates. On the other hand, HCAP is a hydrophilic fluorescent dye that is soluble in water. As a result, the AIE property of HCAP was evaluated by the addition of different fractions of THF in its water solution. Similarly, the fluorescence intensity of HCAP was gradually enhanced as THF fraction increased, and reached the maximum at 95% (Figure 3c,d). The results revealed that both HCA and HCAP were AIE-active.

### 2.2. Titration with ALP

**Figure 4:**
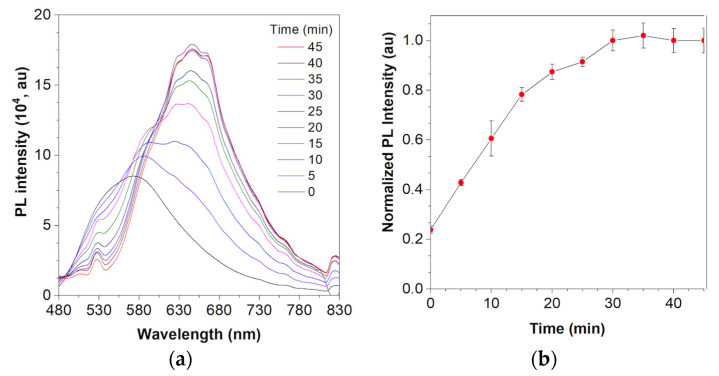
ALP response of HCAP. (**a**) Plot of fluorescence intensity versus incubation time in the presence of 100 mU/mL ALP and 100 μM HCAP in Tris-HCl buffer (pH 9.2). (**b**) Plot of relative PL intensity (I/I_0_) at 645 nm versus the incubation time. I_0_ = PL intensity at time = 0 min.

HCAP is an ALP-responsive probe. In the presence of ALP, the phosphate group on the HCAP probe is hydrolyzed to form HCA. Because HCA is insoluble in water, it forms aggregates and hence gives strong red fluorescent signals. To test the ALP activity of the HCAP, 100 mU/mL ALP was co-incubated with 100 µM HCAP in Tris-HCl buffer (pH 9.2) at 37 °C. The fluorescence spectra were measured every 5 min and collected at 480–830 nm with an excitation wavelength of 430 nm. As shown in Figure 4a, the emission maximum of HCAP was 580 nm at t = 0 min and gradually decreased with time, while the peak at 645 nm gradually increased with time and became steady at 30 min, indicating the formation of HCA aggregates (Figure 4b).

### 2.3. Optimization of Detection Conditions

**Figure 5:**
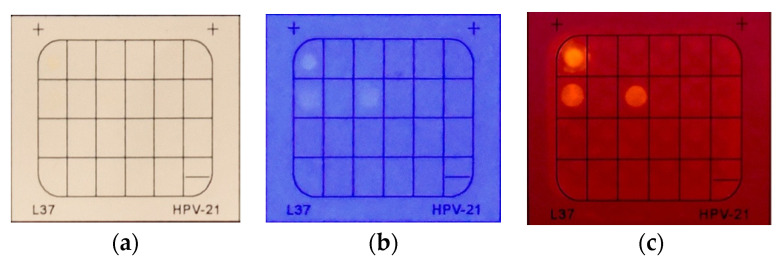
Photography of the HybriMem stained with HCAP in the standard condition of protocol, showing (**a**) daylight, (**b**) 395 nm LED flashlight, and (**c**) 395 nm LED flashlight with optical bandpass filter (600–700 nm). [HCAP] = 0.1 mM; probe incubation time: 5 min; PCR cycle: 40.

Next, we attempted to apply HCAP to the gene detection platform. The results were evaluated by comparing the brightness and clearness of the fluorescent spot verse the background. After the staining procedure, a 395 nm flashlight was used as the excitation source to observe the fluorescent results. As shown in Figure 5, the preliminary result yielded by HCAP was consistent with the BCIP/NBT control group. However, the reflection of the light source and the blue emission of the membrane material caused strong background noise which seriously affected the photograph taken by the camera. Therefore, an optical filter with a wavelength range of 600 to 700 nm was placed in front of the camera lens to block the unwanted background signals.

**Figure 6:**
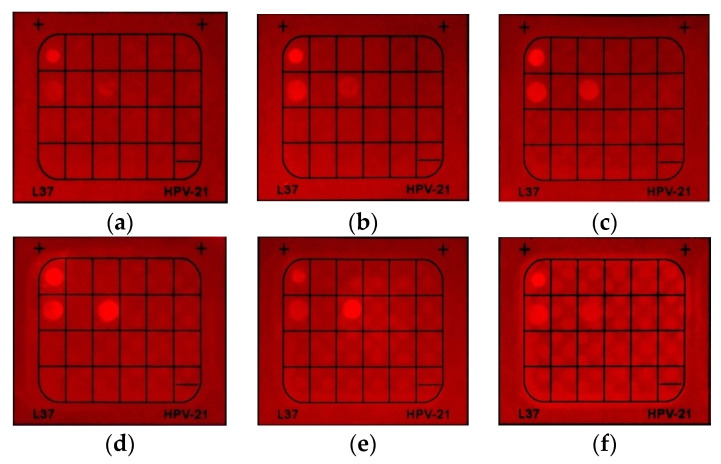
Concentration screening of HCAP, showing (**a**) 10 μM, (**b**) 20 μM, (**c**) 50 μM, (**d**) 0.1 mM, (**e**) 0.2 mM, and (**f**) 0.5 mM. Probe incubation time: 5 min. PCR cycles: 40. Photos were taken under a 395 nm UV lamp with a band-pass filter 600–700 nm.

To further improve the performance of HCAP, several experiments were conducted to investigate the optimal operating concentration and staining time. Regarding the operating concentration, aqueous solutions with concentrations of 10 μM, 50 μM, 0.1 mM, 0.2 mM, and 0.5 mM were applied to the system. Along with the concentration increase from 10 μM to 0.1 mM, the intensity of the fluorescent spots became clear to observe. Due to the observation of background noise for concentrations higher than 0.2 mM, the optimal concentration was determined to be 0.1 mM (Figure 6).

**Figure 7:**
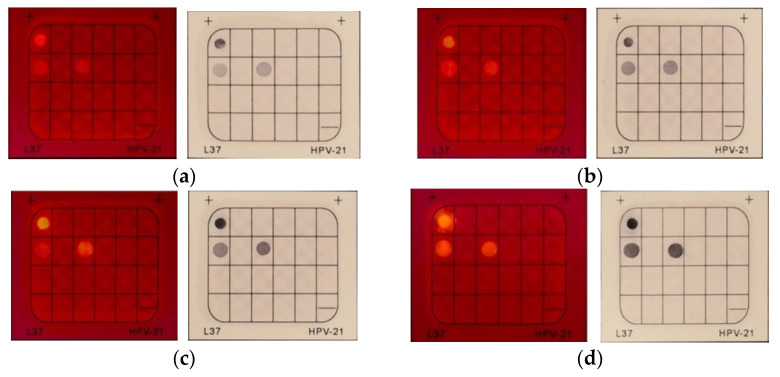
Result comparisons of HCAP and BCIP/NBT on different incubation times, showing (**a**) 30 s, (**b**) 1 min, (**c**) 2 min, and (**d**) 5 min. [HCAP] = 0.1 mM. PCR cycles: 40. Photos were taken under a 395 nm UV lamp with a band-pass filter 600–700 nm for HCAP, while photos were taken under daylight for BCIP/NBT.

Next, the optimization of incubation time was conducted in a similar manner; HCAP solutions of 0.1 mM were applied to the system with the variation in incubation time. (Figure 7). The brightness and clearness of the three fluorescent spots were maintained when the incubation time was reduced from 5 min to 1 min. As the incubation time was further reduced to 30 s, the brightness and clearness of fluorescent spots were significantly diminished. The result indicated that 1 min of incubation time was sufficient to achieve a clear diagnosis result, which is one-fifth of the suggested incubation time of BCIP/NBT. To compare with BCIP/NBT, another set of time screening tests was conducted to determine if a similar trend to that of HCAP would be displayed. BCIP was applied to conduct the same test and the color intensity of the black-purple spots was observed under daylight. As the incubation time reduced from 5 min to 30 s, the black-purple spot intensity gradually dropped. The results revealed that the BCIP signals dropped as incubation time decreased, which trend was comparable to that of HCAP. However, the color of the black-purple spots was significantly diminished when the incubation time was reduced to 1 min. The comparison of 1-min incubation between HCAP and BCIP/NBT demonstrated that signals generated by HCAP were relatively rapid and steady.

### 2.4. Determination of PCR Cycle

**Figure 8:**
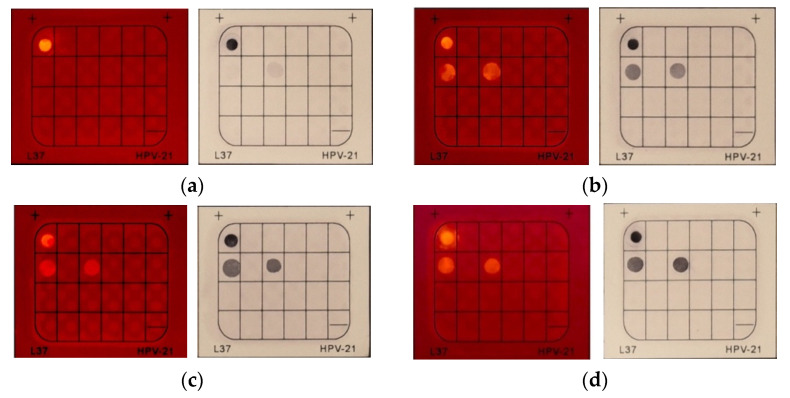
Result comparison of HCAP and BCIP/NBT on the cycles of PCR DNA amplification, showing (**a**) 25 cycles, (**b**) 30 cycles, (**c**) 35 cycles, and (**d**) 40 cycles. [HCAP] = 0.1 mM; probe incubation time: 5 min. Photos were taken under a 395 nm UV lamp with a band-pass filter 600–700 nm for HCAP, while photos were taken under daylight for BCIP/NBT.

The dye with higher sensitivity required a lower amount of DNA for generating a clear signal. The sensitivity tests of HCAP and BCIP were conducted by reducing the DNA applied to the system. To achieve this experiment, PCR cycles of DNA were reduced from 40 to 25, which indicated that a diluted DNA sample was applied to the system. HCAP solutions of 0.1 mM were applied to the system for 5 min of incubation. When the PCR cycles were reduced from 40 to 35 and 30, respectively, the brightness and clearness of fluorescent spots slightly decreased (Figure 8). As the PCR cycle further dropped to 25, only 1 fluorescent spot (biotin control) was displayed on the membrane. The fluorescent spots representing internal control and the positive result (HPV-18) were not observable, which demonstrated that the DNA content was insufficient for hybridization. In the control experiment of BCIP/NBT, when the PCR cycle of DNA amplification decreased from 40 to 30, the color intensity of black-purple spots gradually decreased. However, when the PCR cycle was reduced to 25, the intensity of black- purple spots representing internal control and positive control were significantly diminished. Similar results displayed on the HCAP test and BCIP/NBT control group indicated that HCAP exhibited similar sensitivity to BCIP/NBT. By comparing the results, both HCAP and BCIP/NBT reached their detection limit when the PCR cycle was reduced by 30.

### 2.5. Prototype of the Membrane Reader

**Figure 9:**
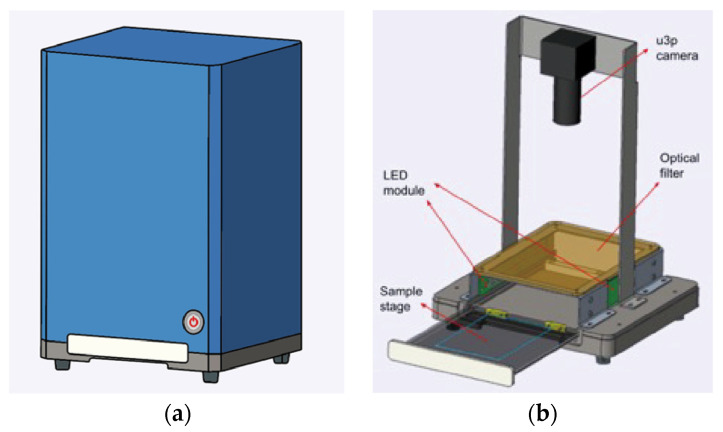
The prototype design of the automated membrane reader. (**a**) Outer appearance of the prototype. (**b**) Interior components of the membrane reader.

A prototype of the membrane reader was developed based on the optimized setting for the HCAP detection system (Figure 9). The result reading was conducted in the dark environment inside the machine, which was fully covered by the metal outer shell. An adjustable sample stage was designed for easy replacement of membranes between different sets of tests. The LED module with a narrow wavelength of 395 nm was installed as the excitation source of HCAP fluorescent signals. An optical filter of 640 nm was used to block excessive background emission from the membrane as well as the strong reflection from the light source. On top of the filter, a U3p camera was installed which was focused on the sample stage to convert fluorescent signals into a digital image. The image was first edited using OpenCV to isolate the individual membrane. Then, the AI software YOLOV5 was used for the automated image analysis. The accuracy of the machine prototype was estimated by comparing the membrane reading result with the traditional BCIP/NBT detection of an identical DNA sample source.

### 2.6. Clinical Trials

**Figure 10:**
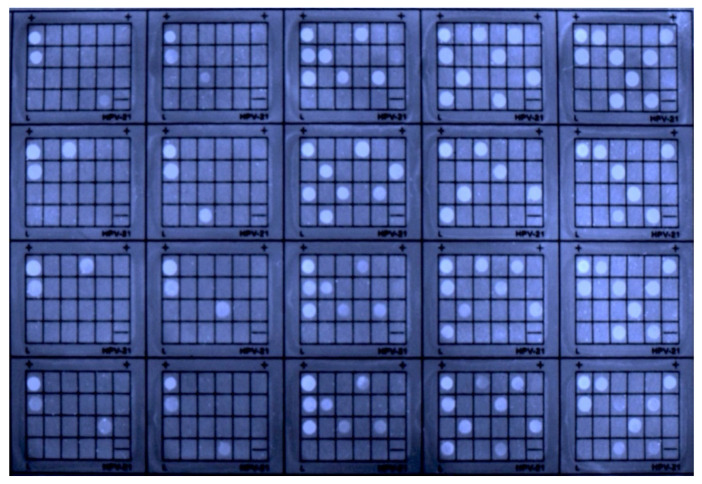
Photographs of the HybriMem stained with HCAP in the standard condition in the clinical trial. The photo of the automatic membrane reader was taken with a U3P camera. [HCAP] = 0.1 mM; probe incubation time: 5 min; PCR cycle: 40.

We further conducted clinical trials by HCAP and used the prototype for recording the results (Figure 10). A total of 267 clinical samples were collected from hospital patients who were suspected to carry HPV genes. Each patient’s DNA sample was divided into two sets. One set was performed by applying HCAP, and another set was tested with BCIP/NBT as the control experiment. The fluorescent patterns of HCAP and BCIP/NBT patterns were compared correspondingly to examine the concordance rate of positive and negative results. From the clinical testing report by HCAP, 53 samples were found to have HPV genes, while 214 samples were negative results. Compared to the results of BCIP/NBT, the total concordance rate was reported to reach 100%, which demonstrated high accuracy and consistency when applying HCAP on prototype machines for genetic diagnosis. If the usage of HCAP on the prototype is optimized and further explored, for instance, by replacing the current membrane with a low-fluorescent material, the prototype has the potential to be commercialized and perform detection of other genetic diseases.

## 3. Materials and Methods

### 3.1. Chemicals, Bioreagents, and Equipments

The chemicals for syntheses were purchased from Sigma-Aldrich, Meryer, and J&K, and used without further purification. All solvents were distilled, purified, and dried following standard procedures. Alkaline phosphatase (ALP) was purchased from Sigma-Aldrich.

^1^H NMR and ^13^C NMR spectra were recorded on a Bruker ARX 400 NMR spectrometer, using suitable deuterated solvents to dissolve the compounds. Mass spectrometry was performed on a Waters Xevo G2-XS QTof mass spectrometer in ESI mode and a GCT premier CAB048 mass spectrometer in MALDI-TOF mode. UV-Vis absorption spectra were measured on a PerkinElmer Lambda 365 Spectrophotometer. Photoluminescence (PL) spectra were measured with an Edinburgh FLS980 spectrofluorometer.

PCR amplification and denaturation were performed on an Applied Biosystems™ 2720 Thermal Cycler. The PCR and hybridization reagents were provided by Hybribio Ltd. and they were commercially available in the Hybribio’s 21 HPV GenoArray Diagnostic Kit (HBGA-21PKG). The compositions of the hybridization reagents are listed in the Supplementary Materials section.

HybriMax^®^ makes use of the flow-through hybridization technology to identify HPV target DNA using specific probes with gene-chip technology. A low-density macroarray platform provides a rapid, in vitro DNA diagnosis with high sensitivity, specificity, and accuracy. The temperature of the reaction chamber can be adjusted from 25 to 75 °C and the pump rate is 70 ± 20 mL/min.

### 3.2. Syntheses of HCAP and Its Intermediates

The synthesis procedure was based on the method described in the previous report [36].

Synthesis of HCA (4-dimethylamino-2′-hydroxychalcone) (1). To a solution of 4-dimethylbenzaldehyde (12 mmol, 1.79 g) and 2′-hydroxyacetophenone (13.2 mmol, 1.80 g) in ethanol (30 mL), KOH (50 mmol, 2.8 g) in water was added and stirred at room temperature overnight. The reaction mixture was neutralized with dilute HCl until the pH value was adjusted to 7.0. The crude mixture was extracted with dichloromethane three times. The combined organic layer was washed with water and dried over Na_2_SO_4_. After filtration, the solvent was evaporated under reduced pressure. The product of (1) was further purified by silica gel column chromatography using gradient elution with hexane/ethyl acetate. ^1^H NMR (400 MHz, CDCl_3_): *δ* (TMS, ppm) 13.23 (s, 1H), 7.93−7.90 (m, 2H), 7.58−7.56 (m, 2H), 7.47−7.43 (m, 2H), 7.02−7.00 (m, 1H), 6.92 (t, 1H), 6.70−6.68 (d, 2H), 3.05 (s, 6H).

Synthesis of HCAPE (2-(3-(4-(dimethylamino)phenyl)acryloyl)phenyl diethyl phosphate) (2). To a solution of (1), (267 mg, 1.0 mmol) and NaH (80 mg, 2.0 mmol) in freshly distilled THF (20 mL), diethyl chlorophosphate (0.3 mL, 2.0 mmol) was added dropwise under nitrogen. The reaction mixture was stirred at room temperature for 2 h. The resulting solution was quenched with water (2 mL), and the solvent was evaporated under reduced pressure. The crude mixture was extracted with dichloromethane three times. The combined organic layer was washed with water and dried over Na_2_SO_4_. After filtration, the solvent was evaporated under reduced pressure. The product of (2) was further purified by silica gel column chromatography using gradient elution with hexane/dichloromethane. ^1^H NMR (400 MHz, CDCl_3_): *δ* (TMS, ppm) 7.55−7.46 (m, 6H), 7.25−7.22 (d, 1H), 7.06−7.02 (d, 1H), 6.67−6.65 (d, 2H), 4.15−4.12 (m, 4H), 3.03 (s, 6H), 1.24−1.23 (t, 6H).

Synthesis of HCAP (2-(3-(4-(dimethylamino)phenyl)acryloyl)phenyl phosphate) (3). To a solution of (2), (201 mg, 0.5 mmol) in dry DCM (25 mL), iodotrimethylsilane (0.28 mL, 2.0 mmol) was added dropwise under nitrogen at 0 °C. The reaction mixture was stirred for 4 h at room temperature before the addition of MeOH (2 mL). After the mixture was stirred for another 0.5 h, the solvent was removed under reduced pressure. The crude product was purified by silica gel chromatography with acetonitrile/water to obtain the compound. The product was freeze-dried overnight after most of the solvent residue was removed under reduced pressure. ^1^H NMR (400 MHz, CD_3_OD): δ (TMS, ppm) 7.77−7.74 (d, 2H), 7.62−7.52 (m, 4H), 7.44−7.38 (m, 2H), 7.28 (t, 2H), 3.02 (s, 6H). HRMS for C_17_H_18_NO_5_P: m/z: [M]^+^, calculated 347.0923, found, 267.1277 [M-PO_3_H_2_]^+^.

### 3.3. Preparation of DNA Samples from Patients

The DNA sample was collected from the cervical orifice of patients by using a sampling brush. The brush was then placed inside the bottom of a sealed bottle which contains the cell preservation solution by the sampling personnel. After sending the sample to the testing center, the 500 μL patient sample was stirred using a vertex mixer, transferred to a centrifuge tube, and centrifuged for 1 min at 14,000 rpm, then the supernatant was removed. For multi-blood samples, the stirring process was prolonged. After the centrifugation, the supernatant was removed and 500 μL sterile purified water was added to rinse the pellet. For mucus samples, the stirring process was prolonged, and the sampling brush was removed from the sample tube before the transfer and centrifugation of the sample. After the brief purification of the sample, 400 μL of pre-warmed solution I (containing Tris, NaCl, EDTA, NaOH, SDS, and purified water) was added to the pellet, stirred by a vertex mixer, and heated to 100 °C for 15 min. After brief centrifugation, 400 μL of solution II (isopropanol) was added to the mixture and stirred using a vertex mixer, then the sample was placed at room temperature for 2 min. Next, the sample tube was centrifuged for 5 min at 14,000 rpm; the supernatant was removed as much as possible. After resting for 2 min, 400 μL of solution III (sterile purified water) was added to fully dissolve the pellet. After resting for 10 min, the sample was centrifuged for 5 min, then the supernatant was collected and ready for DNA amplification.

### 3.4. DNA Amplification by PCR

A volume of 23.25 μL of PCR mix, 0.75 μL DNA Taq polymerase (5U/μL), and 1 μL of DNA sample were added into each PCR tube then mixed via pipetting and vortex mixer. The mixed PCR tube was placed in the thermal cycler for amplification as the programs showed in the Supplementary Materials section. After the denaturation process, the PCR tube was transferred to the Eppendorf PCR cooler for cooling immediately before performing hybridization.

### 3.5. DNA Hybridization

Hybridization solution (containing 0.1% SDS solution), solution A (containing Tris-buffer and 0.05% sodium azide), and solution B (<0.5% SDS solution) were pre-warmed in a 45 °C water bath before the experiment. The HPV-21 HybriMem membrane was installed in the HybriMax^®^. The temperature of the HybriMax^®^ was set to 45 °C. When the temperature was reached, pre-warmed hybridization solution was added to the membrane and allowed to react for 3 min. Then, the solution was pumped off and the hybridization solution was re-added to the well. After that, the denatured DNA sample was added to the well and carefully mixed by pipetting. The solution mixture was incubated for 20 min. After the incubation, the solution mixture was pumped away and washed twice with the hybridization solution; the solution was repeatedly added and pumped away during the washing steps. Next, the temperature of the HybriMax^®^ was set to 25 °C. When the temperature was reached, the blocking solution (containing PBS and <0.1% detergent) was added to the well and incubated for 5 min. After the removal of the remaining solution by pumping, the enzyme conjugate solution (containing < 0.0003% streptavidin–ALP conjugate and stabilizer) was added to the well and incubated for 3.5 min. After the removal of the enzyme conjugate solution by pumping, the membrane was washed with solution A four times; the solution was repeatedly added and pumped away during the washing steps. Then, the temperature of the HybriMax^®^ was set to 36 °C. When the temperature was reached, 0.8 mL of HCAP solution (0.1 mM) was added to the well and incubated for 5 min. After the removal of the HCAP solution by pumping, the membrane was washed with solution B twice and distilled water once; the solution was repeatedly added and pumped away during the washing steps. The membrane was dried by excess pumping and placed in a dark room for photographing.

### 3.6. Spectrophotometric Experiments

Stock solutions (10 mM) of HCA in THF and HCAP in water were first prepared. Then HCA and HCAP were further diluted by THF and water to a concentration of 10 µM. The absorption spectra were recorded from 200 to 800 nm and the fluorescence spectra were collected at 480–830 nm with an excitation of 430 nm.

## 4. Conclusions and Future Perspectives

In this work, we demonstrated that HCAP could be applied to Hybribio’s HPV detection platform as a substitute for the conventional colorimetric system of BCIP/NBT. Fluorescent spots with strong emission and low background were obtained after simple optimization. Compared to BCIP/NBT, HCAP demonstrated diagnostic results with enhanced intensity and clearness at short staining time and low DNA concentration. By taking advantage of the fluorescent signals, an automatic membrane reader was constructed with the deep learning neural network to perform machine learning diagnosis. Additionally, clinical trials of 267 samples were conducted to demonstrate the accuracy and consistency of the prototype. The test result was consistent with the BCIP/NBT control group with a total concordance rate of 100%. The experimental result implied the great potential of the HCAP-based system in detecting other genetic diseases. The design of the prototype and membrane material could be optimized for the fluorescent signal to further improve the efficiency and performance of the system.

## Data Availability

Not applicable.

## Supplementary Materials

The following supporting information can be downloaded at: https://www.mdpi.com/article/10.3390/molecules27207036/s1, Figure S1: The general workflow of the HPV genotype detection platform based on aggregation-induced emission (AIE) and flow-through hybridization technologies; Figure S2: Synthetic route of HCAP; Figure S3: The image of HybriMem in the 21 HPV GenoArray Diagnostic Kit; Figure S4: Schematic diagram of training the deep learning neural network for the machine learning diagnosis of the automatic membrane reader; Figure S5: 1H NMR spectrum of HCAP in CD3OD; Figure S6: The mass spectrum of HCAP; Table S1: Amplification Program’ Table S2: Denaturation Program; Table S3: Composition of Reagents.

## Sample Availability

Samples of the compounds upon request from the authors.
