# Supplementary material for: Development of an HPV Genotype Detection Platform Based on Aggregation-Induced Emission (AIE) and Flow-Through Hybridization Technologies
Source: Molecules. 2022 Oct 18;27(20):7036. doi: 10.3390/molecules27207036 (PMC9609701; doi:10.3390/molecules27207036)
Supplement: Supplementary file 1 [file molecules-27-07036-s001.zip › molecules-1898759-supplementary.pdf]

molecules

MDPI

*Supplementary Materials*

# Development of an HPV Genotype Detection Platform Based on Aggregation-Induced Emission (AIE) and Flow-Through Hybridization Technologies

**Chun-Ho (Charlie) Ma [1], Liejun Li [2], Shuheng Cai [2], Pei Lin [2], Wing-Ki (Kristy) Lam [1], Tsz-Him (Ronald) Lee [1], Tsz-Kin (Ryan) Kwok [1,*], Longxu Xie [2], Tit-Sang (Tom) Kun [2,3,*] and Ben-Zhong Tang [1,4,*]**

[1] Department of Chemical and Biological Engineering, Department of Chemistry, Hong Kong Branch of Chinese National Engineering Research Center for Tissue Restoration and Reconstruction, The Hong Kong University of Science and Technology, Clear Water Bay, Kowloon, Hong Kong, China
[2] Guangzhou Hybribio Biotech Limited, No 71, Fenghuang 3rd Road, Sino-Singapore Guangzhou Knowledge City, Guangzhou 510000 China
[3] Hybribio Limited, Strand 50, Bonham Strand, Sheung Wan, Hong Kong, China
[4] School of Science and Engineering, Shenzhen Institute of Aggregate Science and Technology, The Chinese University of Hong Kong, Shenzhen 518172, China
* Correspondence: chryan@ust.hk (T.-K.K.); tskun@hybribio.cn (T.-S.K.); tangbenz@cuhk.edu.cn (B.-Z.T.)

**Table S1.** Amplification Program.

| Stage | Temperature | Time Period |
|---|---|---|
| Initialization | 95 °C | 9 min |
| | 95 °C | 20 sec |
| Cycle (40 rounds) | 55 °C | 30 sec |
| | 72 °C | 30 sec |
| Hold | 72 °C | 5 min |
| | 4 °C | - |

**Table S2.** Denaturation Program.

| Stage | Temperature | Time Period |
|---|---|---|
| Initialization | 95 °C | 5 min |
| Hold | 95 °C | - |

**Table S3.** Composition of Reagents.

| Reagents | Composition |
|---|---|
| Sample Preservation Solution | Sodium hydrogen phosphate, sodium chloride, purified water |
| Solution I | Tris(hydroxymethyl)aminomethane (Tris), sodium chloride, EDTA, sodium hydroxide, SDS, purified water |
| Solution II | isopropanol |
| Solution III | Sterile purified water |
| PCR Mix | Tris-HCl buffer, magnesium chloride, >3% dATP, dCTP, dGTP and dTTP,<3% synthetic oligonucleotide primers, <0.2 % non-infectious plasmid DNA (for internal control) |
| DNA Taq Polymerase | DNA Polymerase (5U/μL), stabilizer |
| Positive Control | Tris-HCl buffer, EDTA, <0.01% non-infectious plasmid DNA (HPV-18) |
| Hybridization Solution | 0.1% SDS solution |
| Solution A | Tris-buffer, 0.05% sodium azide |
| Solution B | <0.5% SDS solution |
| Blocking Solution | PBS, <0.1 detergent |
| Enzyme Conjugate | <0.0003% Streptavidin-ALP conjugate, Stabilizer |
| BCIP/NBT | 1-5% (w/w) BCIP/NBT |
| HybriMem | Nylon membrane coated with specific HPV DNA probes |

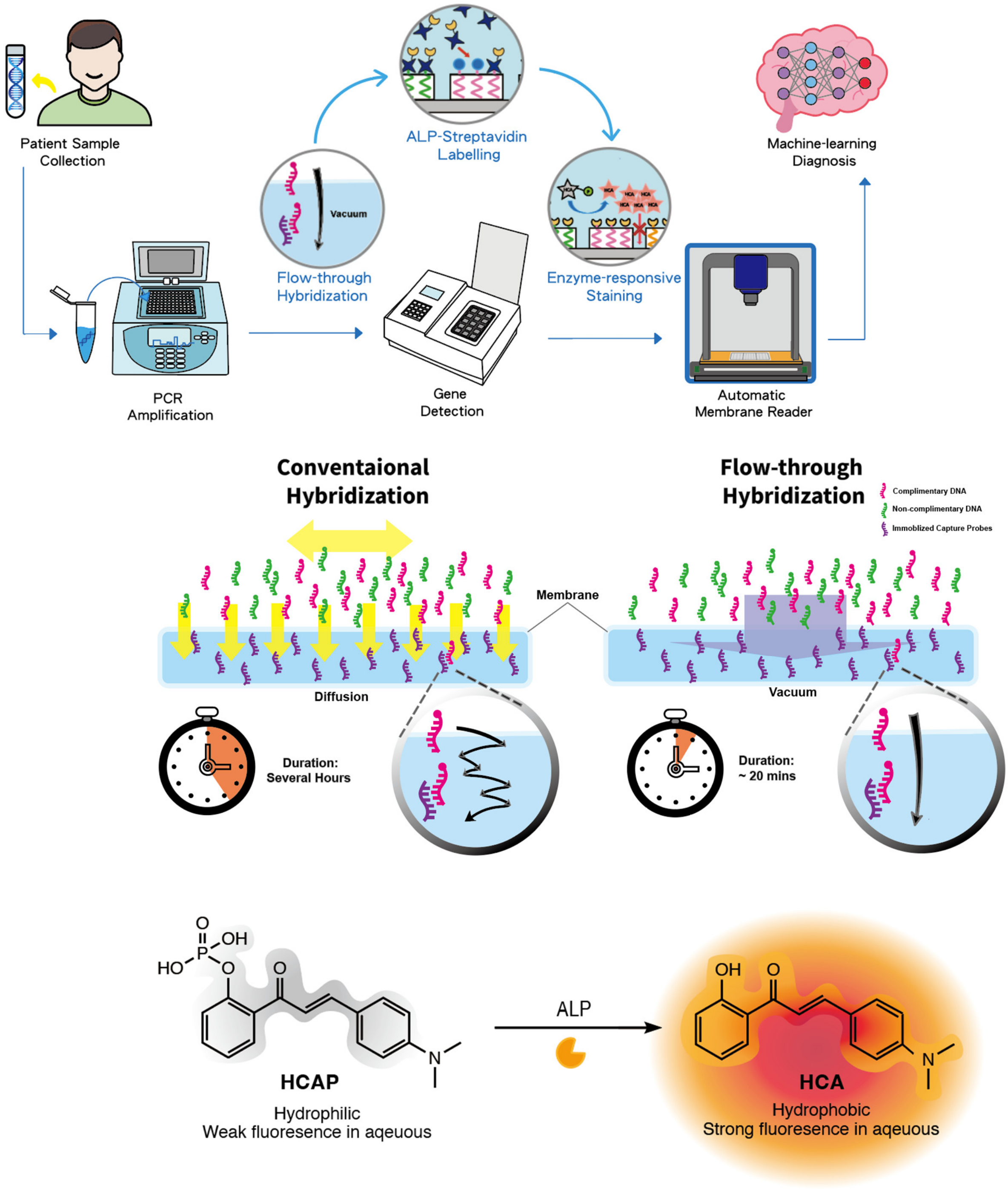


**Figure S1.** The general workflow of the HPV genotype detection platform based on aggregation-induced emission (AIE) and flow-through hybridization technologies.

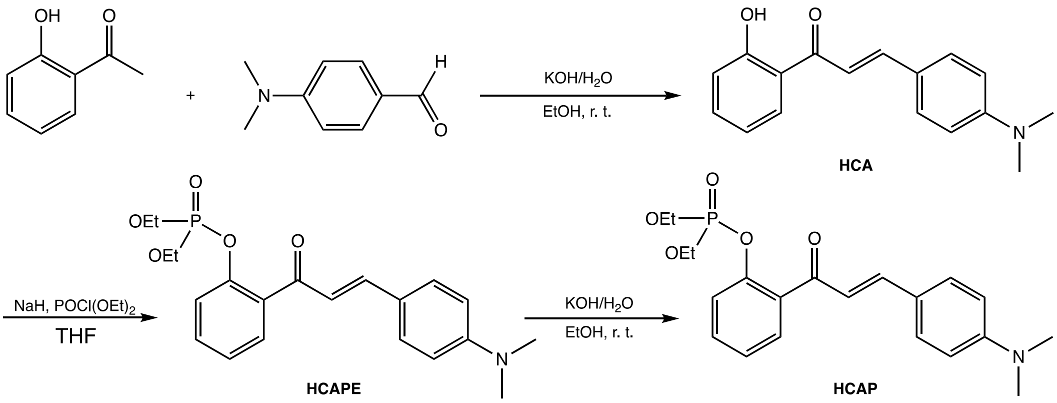


**Figure S2.** Synthetic route of HCAP.

**HybriMem**, is a porous membrane of nylon coated with 21 different high risk HPV type-specific oligonucleotide sequences probes. Capture probes with different oligonucleotide sequences are immobilized on the HybriMem on each array except biotin and internal control.

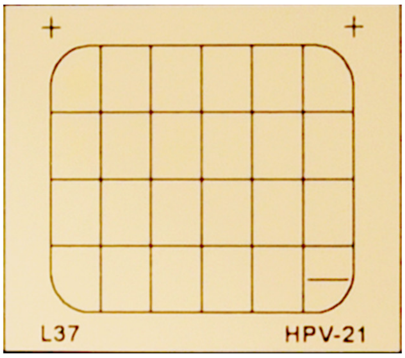


**Figure S3.** The image of HybriMem in the 21 HPV GenoArray Diagnostic Kit.

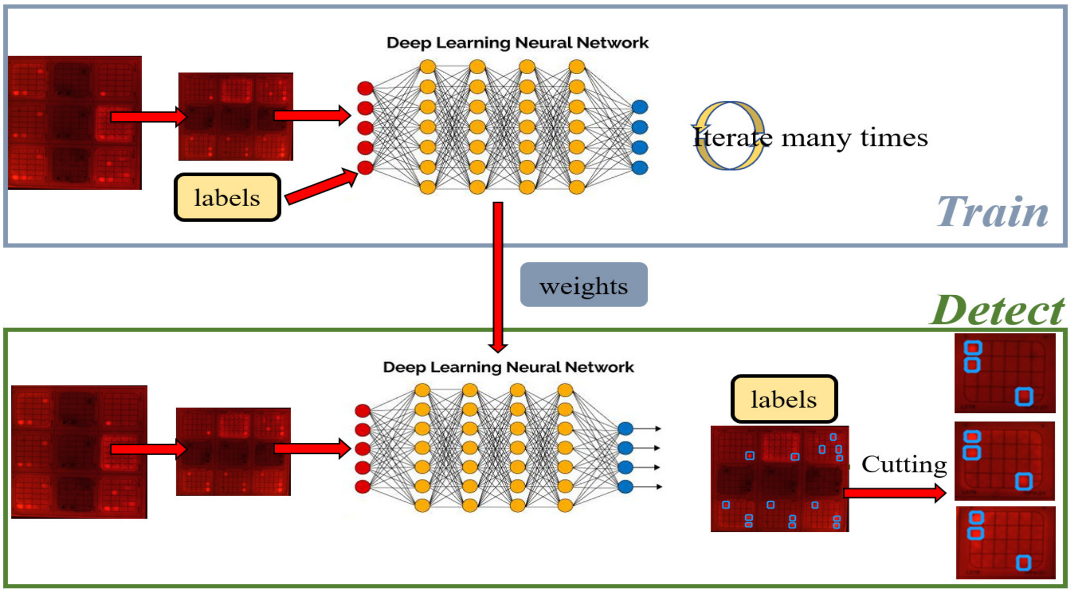


**Figure S4.** Schematic diagram of training the deep learning neural network for the machine learning diagnosis of the automatic membrane reader.

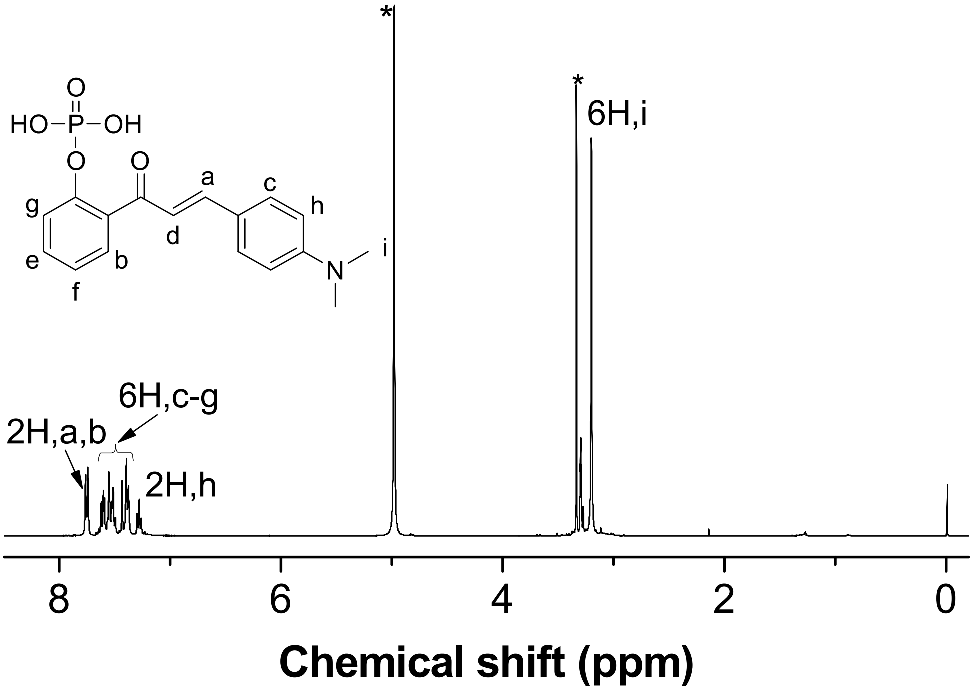


**Figure S5.** $^{1}$H NMR spectrum of HCAP in $CD_3OD$.

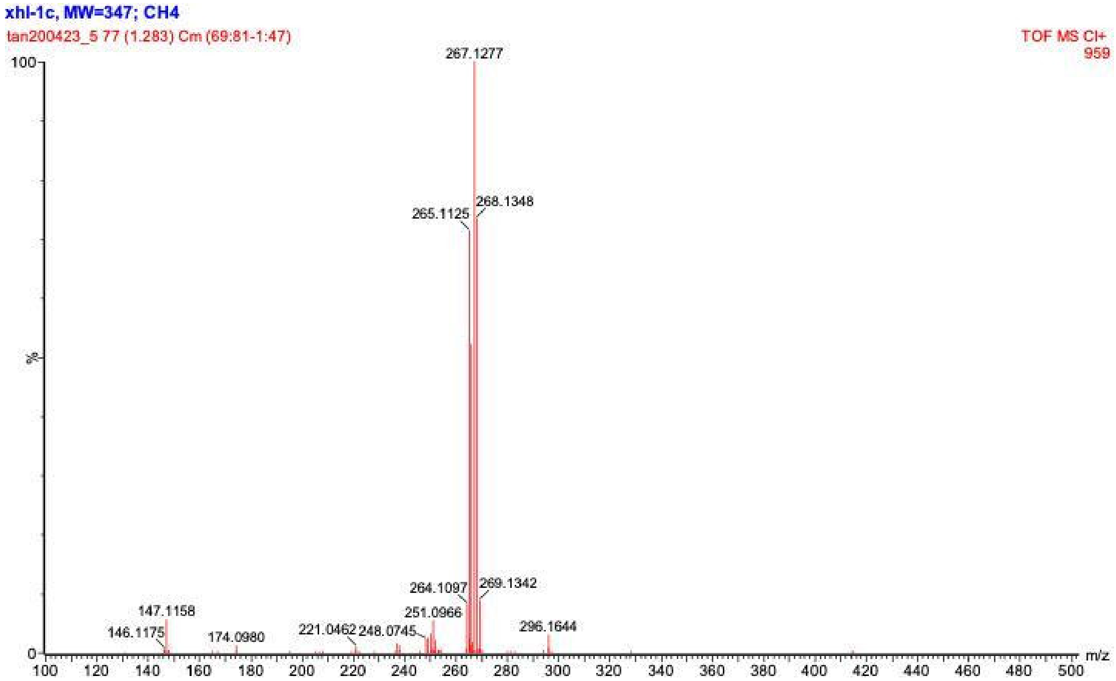


**Figure S6.** The mass spectrum of HCAP.
